# Mr. Hamilton on Epuivocal Generation

**Published:** 1812-01

**Authors:** W. Hamilton

**Affiliations:** Surgeon. Ipswich


*To the Editors of the Medical and Physical Journal.*

GENTLEMEN,

YOUR insertion of a paper signed Medicus, in your Number for December, induces me to appear again before your readers. I confess I feel much satisfaction at the candid manner in which Medicus states his objection, not particularly to my theory, but to the long exploded doctrine of equivocal generation. I shall, therefore, beg leave to call his attention to the explanation of the antiquated experiments which he has adduced, and particularly recommend an attentive perusal of Buffon's Natural History; where he will find ample proof that the bodies which he saw and describes as *worms*, have no more relation to the particular worms of the human body than to any other class of animals.

I believe Leuwenhoek was among the first who discovered these living bodies, or organic particles, in the male semen, from which he endeavored to prove that they were the the embryos of so many men and women,—a doctrine not more absurd than to allow these particles the power of becoming human worms. The experiments of the Count de Buffon, Mr. Needham, and many others, sufficiently prove that these living organic particles are universally to be found in decoctions or solutions of all animal and vegetable substances, and that they are the consequence of the dissolution of all animated nature.

As to Medicus's second experiment, on which he seems to rest his complete conviction of equivocal generation, it has been made long since, not only with boiled and purified vinegar, but also with jellies, boiled and prepared in many different ways, with a view to destroy these apparently-living bodies, but in vain, as they again appeared with this difference only, that they seemed more minute. To prevent prolixity, I shall not occupy your valuable pages with long quotations, but refer Medicus, as well as any others who wish to be acquainted with the subject, to the 2d vol. of Buffon's Natural History, translated into English by Smellie; where he will find his experiments, with many others, related by that celebrated author. In these experiments, however, he will no where find any proof of these organic particles assuming the form of *worms*, which is the principal mistake into which Medicus has fallen; as he quietly concludes from his experiment, "that worms may be produced in any part of the body by diseased matter being thrown out, and entering into the putrifactive fermentation." The meaning of which I take to be that the dissolution or putrifaction of any of the animal solids or fluids, is attended by the production of these organic particles; but why allow these particles the power of becoming particular worms; for we generally find human, as well as other, worms, preserve a specific form. Before we can subscribe to such doctrine, it must be proved by experiment, that living organic particles have the power of assuming this peculiar form, without any sexual intercourse of parents. Even the energetic mind of Buffon himself, who fancied he saw all nature spring from these organic bodies, does not mention any experiment in proof of their transition into any particular species; although loosely and without his usual candour, he supposes their union in an irregular and promiscuous manner to be the general cause of human worms. This being a mere supposition, without any experimental proof, it is unnecessary for me to attempt its refutation.

As it does not appear that there is any proof or analogy of any animal, or species of animals, however minute, being produced without parents; I see no reason for abating the ovate doctrine, by which is meant not merely the egg, but the embryo or fœtus, in whatever form the parents produce it.

By inserting the above you will much oblige your friend and correspondent, W. HAMILTON,

*Ipswich, December* 5, 1811. *Surgeon.*

